# A comparison of hydrological and topological watersheds

**DOI:** 10.1038/s41598-018-28470-2

**Published:** 2018-07-12

**Authors:** B. Burger, J. S. Andrade, H. J. Herrmann

**Affiliations:** 1 IfB, HIT G23.1, ETH Zürich, Zürich, 8093 Switzerland; 2 Departamento de Física, Universidade Federal do Ceará, Fortaleza, 60451-970 Ceará Brazil

## Abstract

We introduce the hydrological watershed, a watershed where water can penetrate the soil, and compare it with the topological watershed for a two-dimensional landscape. For this purpose, we measure the fractal dimension of the hydrological watershed for different penetration depths and different grid sizes. Through finite size scaling, we find that the fractal dimension is 1.31 ± 0.02 which is significantly higher than the fractal dimension of the topological watershed. This indicates that the hydrological watershed belongs to a new universality class. We also find that, as opposed to the topological watershed, the hydrodynamic watershed can exhibit disconnected islands.

## Introduction

Watersheds separate hydrological basins and are an intrinsic property of landscapes^1–4^. Rooted in geomorphology, watersheds play an important role in water management^5,6^ and are as such connected to topological studies about the water retention capacity^7^. They can also be encountered when defining the borders of countries, as seen in the case of Chile and Argentina^8^ or Switzerland and Italy^9^. While watersheds are directly related to percolation theory^10,11^, they furthermore have applications in medicine^12,13^ and image processing^14,15^.

An interesting property of watersheds is their self-similar character^16^. Fractality is a general, important property of physical structures and in the case of watersheds it can be used to deduce the length of the watershed in dependency of the scale. This can be helpful to correctly estimate the length of watersheds when rescaling digital maps. The fractal dimension of an object is also a measure for its structure, as two objects with different fractal dimensions will scale differently. It can furthermore connect phenomena that are at first glance seemingly unconnected physical problems if they have the same universal fractal dimension^17–20^. For artificial landscapes and for digital elevation models, the fractal dimensions of watersheds have already been calculated under the condition that water only flows on the surface and can not penetrate into the soil, which we will call here the *topological watershed*^21–25^. This watershed is in the same universality class as the optimum path crack^26^, the shortest path on loop-less percolation, polymers in strongly disordered media^27^ and bridge percolation^17^. The topological watershed has been shown numerically to be SLE^28^.

The question we address is how the structure of a topological watershed of a two-dimensional surface changes compared to a hydrological watershed, where water can penetrate the soil. Whether and how the fractal dimension of the watershed changes has not yet been investigated and is the subject of the present paper. To answer this question, we use a generalized version of the invasion percolation (IP) based^29^ algorithm proposed by Fehr *et al*. in ref.^22^. Throughout this paper we use an uncorrelated, artificially generated landscape to investigate the structural changes in a more controllable environment.

## Methods

For the definition of the hydrological watershed’s landscape, we choose a three-dimensional grid consisting of sites *i* with heights *h*_*i*_ on the upper surface and permeabilities *p*_*i*_ below the surface. The heights *h*_*i*_ are chosen randomly between zero and one. In this way, we model different permeabilities of the soil which lead to different penetration depths. For a soil discretized as a cubic lattice with edge length *L*, we implement the penetration-hindrance such that the resistance increases systematically with increasing depth *n*_*i*_. To control the seeping depth of the water, we generate the permeabilities *p*_*i*_ as the sum of a randomly generated part with an offset which depends on the layer *n*_*i*_ of site *i* given by,

1 $${p}_{i}=r+a{n}_{i},$$

where *r* is a random number homogeneously distributed between 0 and 1, *a* is the parameter of the model which controls the depth the water reaches, and *n*_*i*_ is the layer of site *i* divided by the edge length *L* which also yields a number between 0 and 1.

The *outlets* of a terrain can be something like a river, underground river, a lake, or any other structure in which water can leave the system. We choose the outlets to be two opposite, vertical sides of the cube. Helical boundary conditions are implemented on the two non-outlet, vertical sides of the cube in order to make sure to keep the water inside the system, if it is not leaving at one of the two outlets. The helical boundary conditions on the non-outlet surfaces are implemented such that the neighbors of site *i* of a three-dimensional lattice stored in an array are given by the sites *i* − 1, *i* + 1, *i* − *L*, *i* + *L*, *i* − *L*^2^, *i* + *L*^2^. We do not have to care about the bottom boundary if we choose the depth of the cube to be greater than ${d}_{min}=\frac{1}{a}$. The water will never reach a layer larger than *d*_*min*_ because the permeability ${p}_{{d}_{min}}$ represents the maximum possible permeability of the upper surface *h*_*max*_ = 1 and, therefore, acts as a boundary for the seeping water.

The most important aspect for extracting the watershed is to find out to which outlet the water at site *s* drains to, thus to find in which *catchment basin s* lies. For this we follow a procedure that is called *growing an IP cluster*^22^, where IP stands for invasion percolation^29^. *p* is the permeability for sites that are below the surface as well as the height of sites that are on the surface. Starting at *s*, the water takes the path of steepest descent. We denote the *i*^*th*^ site in the IP cluster with *c*_*i*_. At each step $i > 1$, we add the neighbor of site *c*_*i*−1_ with the smallest ${p}_{{c}_{i-1}}$ to the cluster, but only if the *p*_*neighbor*_ is smaller than ${p}_{{c}_{i-1}}$ and if it is not already belonging to the cluster. If for all neighbors *p*_*neighbor*_ is larger than ${p}_{{c}_{i-1}}$, the cluster is stuck in a local minimum that we call a *pore*: A pore consists of all sites *p* that have the same *p* as *c*_*i*−1_ and is the equivalent of a lake in the case of a topological watershed. We flood the pore and add the neighbor *n*_*min*_ with the lowest permeability on its perimeter to the cluster, thus we set *c*_*i*_ = *n*_*min*_. By repeating this procedure we will at the end always reach an outlet. We know that all sites of the same IP cluster drain to the same outlet and we thus label all sites *c*_*i*_ within one IP cluster as belonging to the same catchment basin. However, the procedure of growing IP clusters alone is not yet efficient if we grow them from each site of the surface. It is more convenient to start with one segment of the watershed. We label each adjacent site with the outlet to which the water flows by growing an IP cluster. The next segment of the watershed then comes to lie between the two adjacent sites that drain to different outlets of the system. By repeating this procedure we grow the watershed until it spans the system from one side to the other. The initial segment of the watershed can be found by moving from one outlet of the system to the other while growing IP clusters.

### Data availability

The datasets generated during and/or analyzed during the current study are available from the corresponding author on reasonable request.

## Results

**Figure 1:**
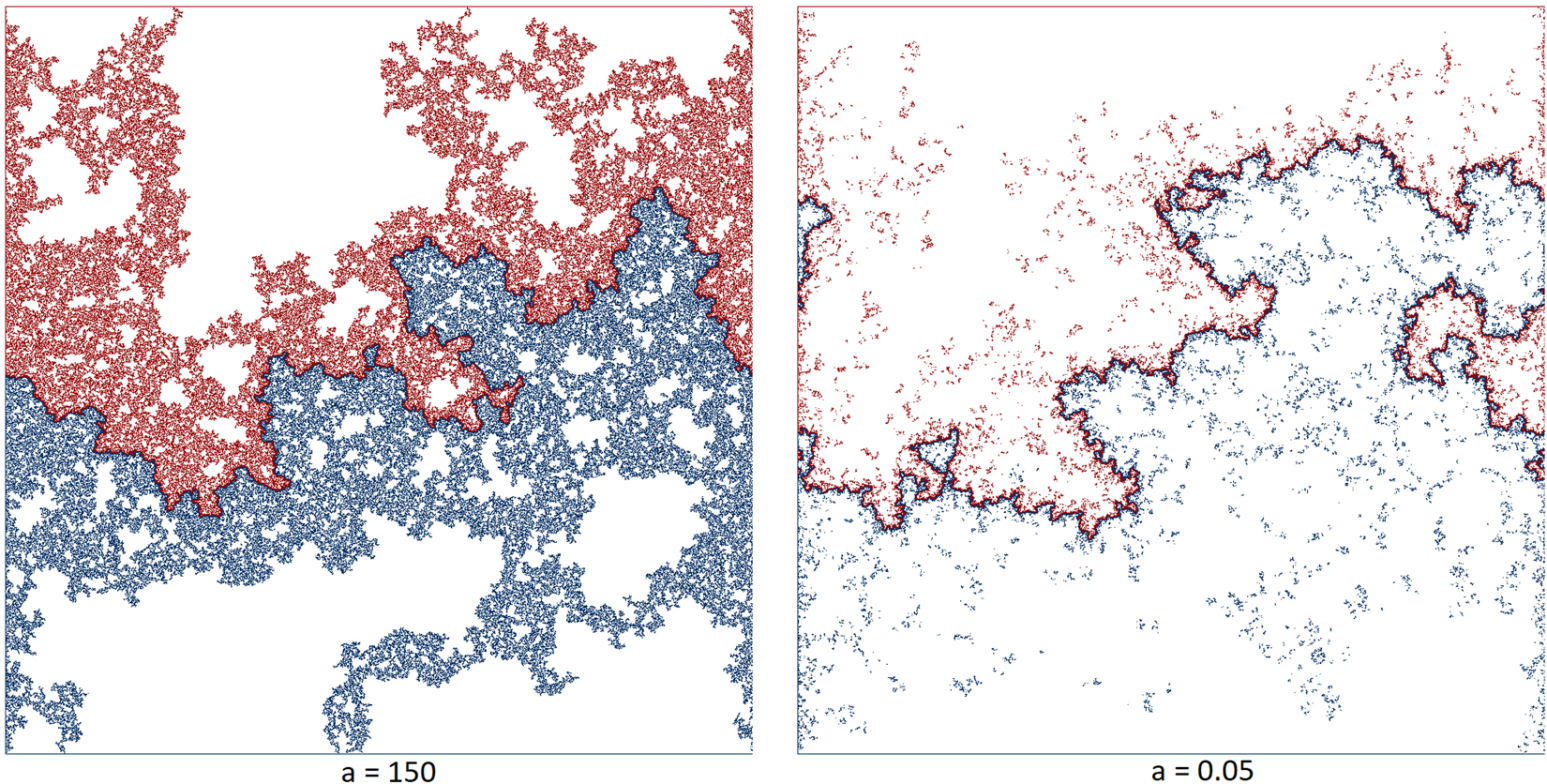
The dark line is the watershed extracted with the IP-based algorithm for three-dimensional models generated according to Eq. (1) for different *a* and grid length *L* = 1000. The red points belong to an IP-cluster that drains to the upper basin and the blue ones belong to an IP-cluster that drains to the lower basin. The figure was created by storing the top layer of the system as a bitmap.

The comparison between the landscape of a hydrological and a topological watershed shows that on the top layer of the hydrological case, less sites belong to an IP-cluster than for the topological case (see Fig. 1). This is a direct result of the penetration resistance. For $a\ll 1$, a large part of the IP-cluster’s mass exists underground, whereas for $a\gg 1$, the water mostly stays at the surface, since its room for evasion into the soil is very small.

**Figure 2:**
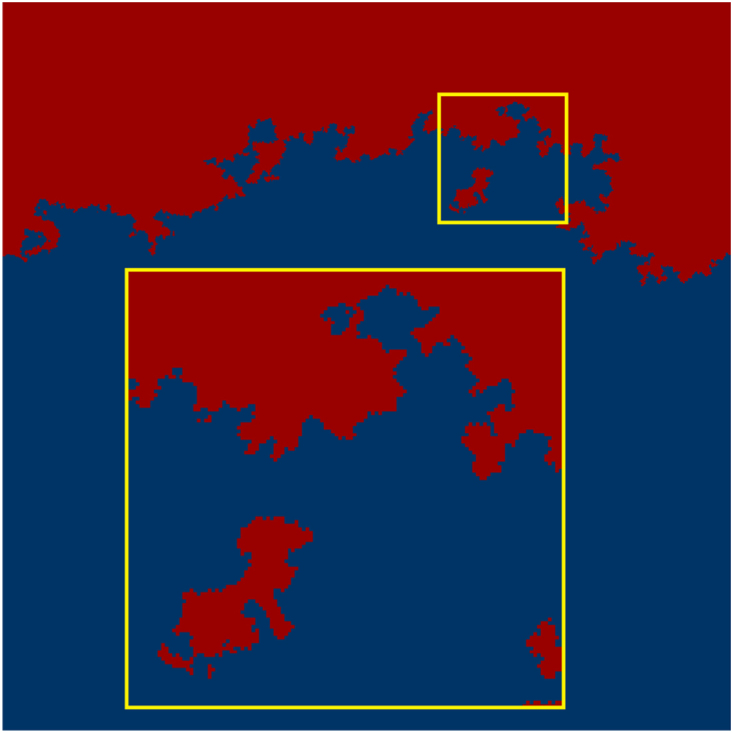
The top layer of a typical realization of a hydrological watershed, calculated for *a* = 0.05 and a grid length of *L* = 1100. Colors as in Fig. 1. The figure was created by storing the top layer of the system as a bitmap.

We find that as opposed to the topological watershed, the hydrological watershed is not always a connected line anymore, as islands can occur (see Fig. 2). An island is a region of connected sites belonging to a catchment basin *i* that is surrounded by sites belonging to catchment basin *j* ≠ *i*. In Fig. 2 we can even observe the formation of islands within an island. We surmise that the size of these islands and their relative placement to the watershed depends on the penetration depth of the water, or, expressed equivalently, on the extent of three-dimensionality of the landscape. The size of the islands as well as the distance from the watershed increases with decreasing penetration resistance.

Using the yardstick method^30^ we measured the fractal dimension of the watershed for each simulated system, where the heights *h*_*i*_ and the permeabilities *p*_*i*_ are generated with a congruential number generator^31^. For each system *i*, we get a data set consisting of tuples containing the stick length *ε* and the number of sticks *N*(*ε*) needed to approximate the watershed. The function *N*(*ε*) satisfies $N(\varepsilon )\propto {\varepsilon }^{-{d}_{f,i}}$, where *d*_*f*,*i*_ is the fractal dimension of the watershed of system *i*. For each value of *a*, we average the fractal dimension over *N* landscapes.

**Figure 3:**
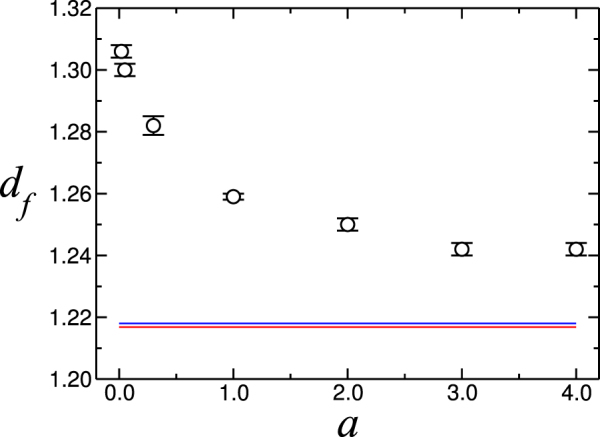
Measured fractal dimensions as a function of the depth parameter *a*. The blue line represents the value for *a* = 1200, while the red line represents the topological watershed corresponding to the asymptotic value *a* → ∞, as reported by Fehr *et al*. in^22^.

The results obtained for the fractal dimension show that for the hydrological watershed, the fractal dimension decreases with the model parameter *a* (see Fig. 3). From these data we linearly extrapolated the fractal dimension of the three smallest measurement values to the limit *a* → 0 and found it to be 1.31 ± 0.02. We obtained the error bar by finding the lines of maximum and minimum slope that still fit the data. For *a* → ∞ the fractal dimension of the topological watershed is consistent with the high precision calculation by Fehr *et al*., namely, 1.2168 ± 0.0005^24^.

**Figure 4:**
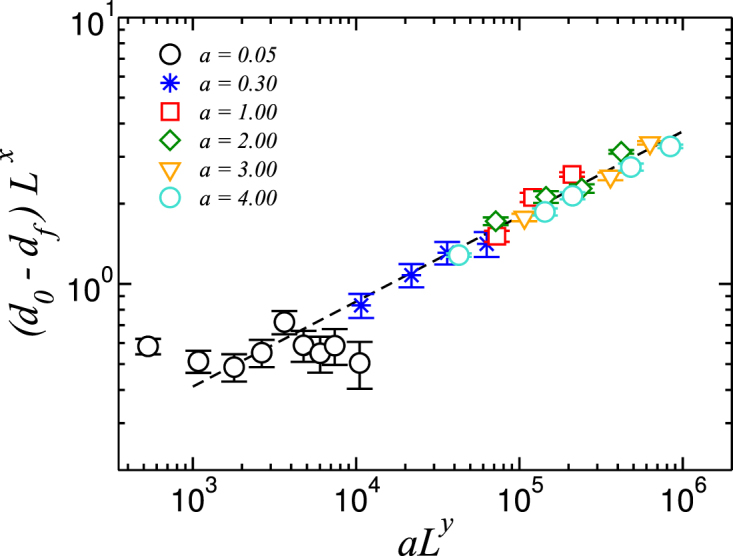
Scaling function for different parameters *a* and sizes *L*. The dashed line is a guide to the eye of slope −*x*/*y*.

Previous measurements of the fractal dimension of a watershed surface in three dimensions yielded a fractal dimension of 2.487 ± 0.003^24^. One might naively identify the hydrological watershed as the cut between this three dimensional watershed with the surface of the landscape. Therefore, we could expect the hydrological watershed to have a fractal dimension of 2.487 − 1 = 1.487. In what follows, we investigate the possibility that it might be possible that our numerical extrapolation *d*_*f*_ = 1.31 ± 0.02 is only part of a crossover, with the hydrological watershed asymptotically having fractal dimension *d*_*f*_ = 1.487. For this purpose, we analyzed the finite size scaling behavior of the watershed. The ansatz for the scaling function is given by,

2 $${d}_{0}-{d}_{f}={L}^{-x}F(a{L}^{y}),$$

where *d*_0_ denotes the fractal dimension for *a* = 0 and *L* → ∞, and *x*, *y* are scaling exponents. For our analysis, we chose to evaluate the scaling law for both *d*_0_ = 1.31 and *d*_0_ = 1.487. The scaling exponent *x* can be obtained using the relation, *d*_0_ − *d*_*f*_ ∝ *L*^−*x*^, when setting *a* = 0. Assuming the same relation for *a* = 0.05, we get a first estimate for *x*. Then, plotting Eq. 2 as shown in Fig. 4, we obtain final values for *x* and *y* through the best data collapse for different *a* and *L* with the constraint that the slope in a log-log plot be −*x*/*y*. By choosing *d*_0_ = 1.487, such a data collapse only becomes possible for very unlikely exponents, namely *x* being either 0.05 or *y* being 20. Setting *d*_0_ = 1.31, yields *x* = 0.56 ± 0.05 and *y* = 1.75, with a convincing data collapse (see Fig. 4). In this way, no realistic scaling function can be found for *d*_0_ = 1.487, whereas for *d*_0_ = 1.31 we observe data collapse for a reasonable choice of *x* and *y*. This suggests that we are not facing a crossover to the fractal dimension *d*_*f*_ = 1.487, but we have discovered, in fact, a new universality class for the hydrological watershed.

## Discussion

The topological watershed which is directly obtained from the discrete elevation map has been studied extensively in the past^21–24,32^. We found that the penetration of water into the soil not only modifies the topological watershed, but also changes its continuity, and even its fractal dimension. The value of the fractal dimension of the topological watershed in a two-dimensional model is known to be 1.2168 ± 0.0005^24^. Our measurements for the fractal dimension show that the fractal dimension of a hydrological watershed is *d* = 1.31 ± 0.02. Comparing this to the fractal dimension of a watershed surface in three dimensions and its fractal dimension of 2.487 ± 0.003^24^, we first expected to obtain a fractal dimension of 1.487. We ruled out the possibility of our data being part of a crossover with a scaling analysis, which showed convincing data collapse for *d*_*f*_ = 1.31 ± 0.02, but not for 1.487 ± 0.003. We can therefore assert that the hydrological watershed belongs to a new universality class.

We obtained the measured fractal dimensions for a model of randomized linear permeability in uncorrelated soil. Our permeability model could very well be substituted with another soil generation method that allows for variations over the penetration depth of the water and which would be equally justifiable. Furthermore, a new soil generation method could account for spatial correlations that are occurring in real soils, which are described by a Hurst exponent *H*^23,33^. It is relatively easy to extend our algorithm to a different soil generation method as it does not affect our watershed calculation method and measurement methodology.
